# Case Report: Baricitinib improved alopecia areata in a pediatric patient with atopic dermatitis

**DOI:** 10.3389/fped.2024.1497285

**Published:** 2025-01-10

**Authors:** Sihan Wang, Ziqian Xu, Xiaoxia Zhu, Xin Fan, Yingzhe Yu, Bingjiang Lin, Suling Xu

**Affiliations:** Department of Dermatology, The First Affiliated Hospital of Ningbo University, Ningbo, Zhejiang Province, China

**Keywords:** alopecia areata, atopic dermatitis, JAK inhibitors, baricitinib, pediatric

## Abstract

Alopecia areata (AA) is a common non-scarring hair loss condition whose specific pathogenesis is not yet fully understood. In children, AA often co-occurs with atopic dermatitis (AD), complicating treatment. Here, we report the case of a child with myasthenia gravis who had severe AA and moderate AD. The child had previously been treated with local injections of corticosteroids and developed total hair loss and AD after discontinuing corticosteroid use. After approximately one year of treatment with baricitinib, 4 mg once daily, combined with twice-daily application of a corticosteroid ointment, a significant improvement in the child's condition was observed, with the Severity of Alopecia Tool score dropping from 100 to 24.4 and Eczema Area Severity Index score to 0. New vellus hairs were clearly observable under trichoscopy, which contrasted significantly with the pre-treatment state. Throughout the treatment process, the patient's clinical symptoms, blood cell counts, liver and kidney function, and coagulation functions were essentially normal, with no significant adverse reactions observed except for folliculitis on the scalp. We discuss common targets in the pathogenesis of AA and AD as well as the safety and prospects of Janus kinase inhibitors for the treatment of pediatric patients with these conditions.

## 1 Introduction

Alopecia areata (AA) is a chronic, relapsing autoimmune disease characterized by non-scarring, round or oval patches of hair loss, with alopecia universalis being the complete loss of all hair and body hair. AA often co-occurs with atopic dermatitis (AD). Both conditions are difficult to cure completely, with severe effects on the quality of life and mental health of patients (1), especially in children. Systemic treatments for moderate-tosevere AD in children over 12 years old include hormones, cyclosporine, antibodies targeting the type 2 inflammatory pathway (dupilumab), and Janus kinase (JAK) inhibitors (baricitinib, abrocitinib, upadacitinib), with only baricitinib being approved by the Food and Drug Administration (FDA), European Medicines Agency (EMA), and China Food and Drug Administration (CFDA) for the treatment of moderatetosevere AA in adults. There have been numerous reports in the literature on the efficacy and safety of baricitinib in children with severe AA in recent years. Here, we report the case of a 14-year-old child with severe AA and moderate AD who was successfully treated with baricitinib.

Informed consent was obtained from the patient and his grandmother for participation in the study and publication of the article, including publication of clinical photographs and laboratory test results.

## 2 Case description

**Figure 1:**
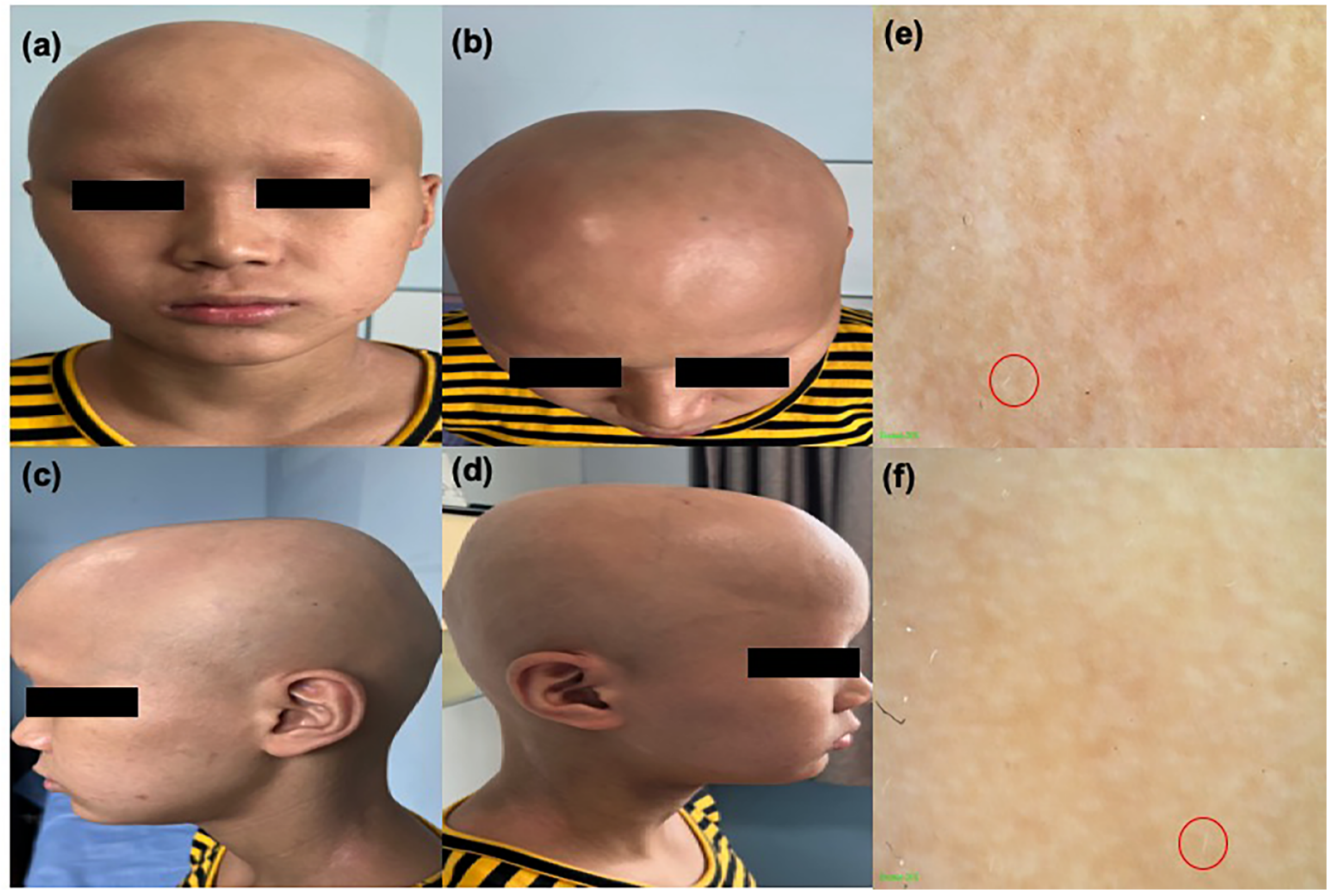
**(a)–(d)** Clinical image of the patient showing alopecia areata. **(e)** and **(f)** Trichoscopy image of alopecia areata; a small number of white vellus hairs can be observed within the red circle.

We report the case of a 14-year-old male, born in southeast China, with a maternal history of allergic rhinitis. The patient had had eczema since the age of 2 years and myasthenia gravis since the age of 5 years. He had undergone high-dose corticosteroid pulse therapy for approximately 2 years at a local hospital. After his myasthenia gravis improved and the medication was discontinued, he began to develop exacerbated eczematous skin lesions all over his body, with the scalp being the most severely affected. The patient found the itching intolerable and repeatedly scratched his scalp. Since the age of 8 years, he had experienced patchy hair loss on both sides of his scalp, which was treated with local injections of corticosteroids. After treatment, his hair regrew, but after discontinuing the medication, all of his hair fell out and parts of his eyebrows and eyelashes were also affected. When the patient came to our department, erythema and scratch marks were visible on his head, face, trunk, and both upper limbs. His skin was dry and itching was significant, with an Eczema Area Severity Index (EASI) score of 18, indicating moderate AD. His hair and eyebrows had almost completely fallen out, his eyelashes were very sparse, and the Severity of Alopecia Tool (SALT) score was 100 (Figure 1), with no nail abnormalities observed, leading to a diagnosis of alopecia universalis. The patient was also quite introverted, avoiding direct eye contact, and wore a mask and hat throughout the visit.

In addition to AA, we considered the possibility of other conditions such as trichotillomania, tinea capitis, scarring alopecia, and telogen effluvium. However, the diagnosis of AA can usually be confirmed based on the characteristic appearance of the bald patches, and the patient's medical history largely ruled out trichotillomania and telogen effluvium. Although the clinical symptoms of scarring alopecia are very similar to those of AA, in this condition, hair follicle openings are not visible upon clinical examination and tinea capitis can be excluded through fungal tests and hair microscopy.

After communicating with the patient and his family (who refused treatment with corticosteroid injections again) and considering the patient's medical history, we decided to treat the AA and AD with baricitinib 4 mg once daily combined with twice-daily application of dinideotide ointment. Baricitinib is currently the only drug approved in China for the treatment of adult AA. It has been approved by the FDA for adult rheumatoid arthritis, AA, and COVID-19, and by the EMA for the treatment of AD in children and adults over 2 years old, adult AA, and juvenile idiopathic arthritis in children over 2 years old. Before treatment, we performed a complete blood cell count, liver and kidney function tests, viral hepatitis markers, and tuberculosis screening. During treatment, complete blood cell counts, tests of liver and kidney function, and coagulation function tests were conducted regularly.

**Figure 2:**
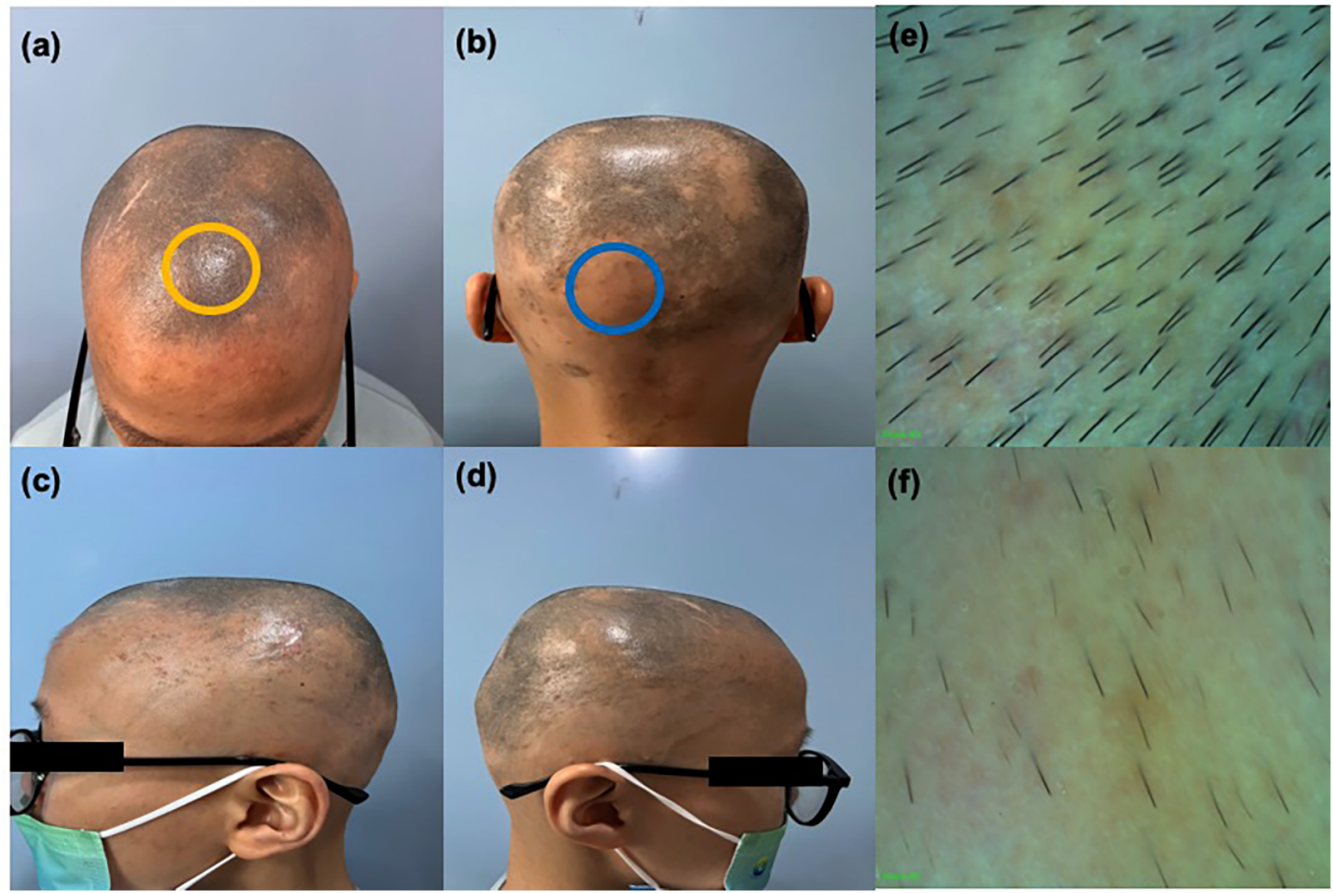
**(a)–(d)** Clinical image of the patient showing alopecia areata after 59 weeks of treatment. **(e)** Enlarged trichoscopy image of the yellow circle **(f)** enlarged trichoscopy image of the blue circle.

In the first 12 weeks of treatment, only a small number of new white vellus hairs could be seen by hair microscopy, and the patient reported being able to feel the new growth. At around 24 weeks of treatment, multiple clusters of short, dark new hairs were visible to the naked eye and the eczematous skin lesions on the patient's trunk and upper limbs had receded. The patient occasionally experienced itching and scaling on the face, which could be relieved with emollients. After 59 weeks of treatment, we observed significant clinical improvements in both the patient's AD and AA (Figure 2), with the SALT score dropping from 100 to 24.4 and the EASI score to 0. Hair microscopy indicated regrowth of hair on the scalp, eyebrows, and eyelashes. The patient's eczematous skin lesions had completely resolved. The patient reported occasional skin itching when exposed to heat, which could be alleviated with antihistamine medication.

Throughout the treatment process, the blood cell counts, liver and kidney function, and coagulation function tests showed no significant abnormalities. Scattered episodes of folliculitis on the scalp occurred during the treatment, which increased noticeably after intense physical activity and sweating; these could be resolved with the application of fusidic acid ointment. At week 52 (during the hot summer months), a large number of folliculitis lesions appeared on the scalp that had not completely resolved by week 59. The patient's scalp folliculitis may have been related to his habit of wearing a hat, as no significant acne or folliculitis were observed on his face, neck, or trunk. The patient's family reported that because of his severe AA, the patient wore a hat every day when going out, including on the way to school, during meals and medical visits, and even during physical activities. The patient was also quite introverted, communicating with us through either nodding or shaking his head, during the early stages of treatment, but began to actively communicate with the doctor at week 24. By week 52, he could independently describe his condition, although he continued to wear a hat consistently outside of treatment sessions. The patient and family are very satisfied with the current treatment effects and are willing to continue the current treatment and follow up regularly.

Currently, the patient is still under follow-up. Our limitations include the lack of histopathological data and long-term follow-up information, especially regarding the potential impact on growth and development in children.

## 3 Discussion

Alopecia areata (AA) is a complex disease resulting from the interplay of multiple factors, and its pathogenesis involves multiple biological targets. The prevailing view is that AA compromises the immune privilege of hair follicles (2); however, the structure of the follicles is not destroyed the damage is mainly to the hair growth cycle (3). In skin biopsies of patients with AA, anagen hair follicles were found to be affected by the infiltration of inflammatory cells—primarily CD8+ T cells and CD4+ T cells, as well as antigen-presenting cells (APC), mast cells, NK cells, and eosinophils—around and within the follicle (4). Studies have shown that the levels of Th2-related markers (IL-13, CCL13, CCL17, CCL22, and CCL26) in the serum of patients with AA are significantly elevated, and the degree of Th2 activation increases with the severity of hair loss (5). Among them, IL-13 and IL-4 both bind to the so-called type II receptor, composed of the IL-4Rα chain and the IL-13Rα1 chain, thereby activating JAK1 and tyrosine kinase 2 (TYK2), which in turn leads to the activation of STAT6. There is literature indicating that dupilumab, which can block the IL-13 and IL-4 pathways exerts therapeutic effects in patients with AA (6).

**Figure 3:**
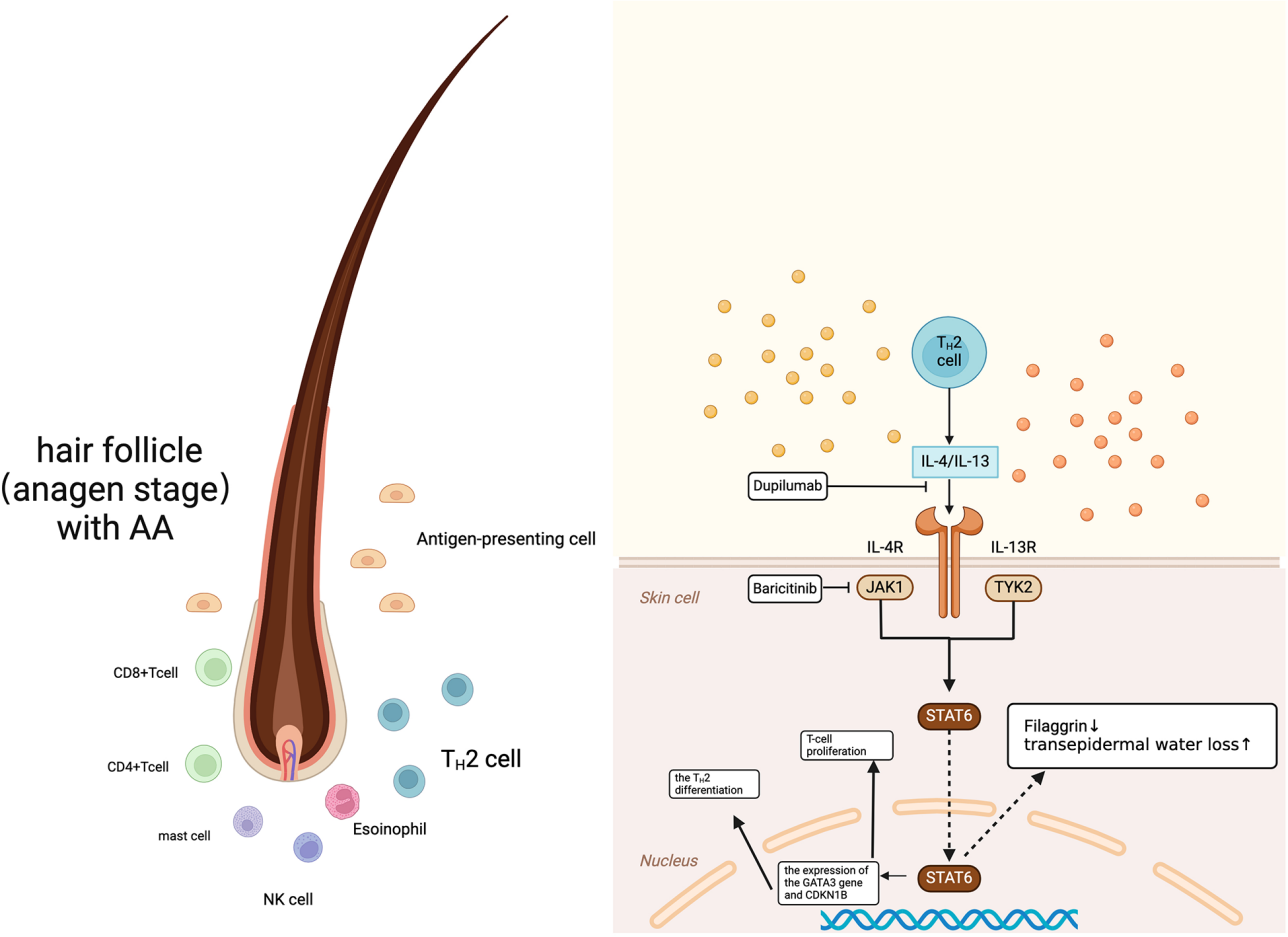
Common immunopathogenesis of alopecia areata AA and AD. Created in BioRender. Wang, S. (2024) https://BioRender.com/g04h745.

During the pathogenesis of AD, Th2 cells expressing chemokine receptorhomologous molecules infiltrate the skin and produce IL-13, IL-31, and IL-4 (7). IL-4 and IL-13 significantly reduce the expression of key structural proteins of the skin barrier (such as filaggrin) by activating STAT6 (Figure 3), leading to an increase in transepidermal water loss that is typically measured, disrupting the integrity of the stratum corneum and exacerbating the severity of AD (8).

Other studies have found that filaggrin (*FLG*) gene mutations are an important risk factor for AD and are associated with the severity of AA (9). Palmer et al.'s research indicates that *FLG* loss-of-function mutations are closely related to AD (10). Regina C Betz et al.'s findings show that *FLG* mutations can drive the severity of comorbid AA in patients with AD. In the absence of *FLG* mutations, the comorbidity of atopic diseases has little or no impact on the severity of AA (11).

Drugs targeting the JAK pathway, such as baricitinib, can achieve therapeutic effectiveness in patients with AA and AD. In this case, owing to severe AA, the patient had been wearing a hat for a long time, which exacerbated his folliculitis. Furthermore, the patient had a relatively introverted personality, which is not uncommon (12). Children with AA and AD tend to have a much higher probability of depression than healthy children. A study by Peła et al. shows that the possibility of AA and AD appearing in patients with depression and in their families is higher. This may be because stress induces the HPA axis to release cortisol, adrenocorticotropic hormone, and beta-endorphins. Acute high levels of stress hormones have an immunosuppressive effect on Th1 cells while mediating the differentiation of T helper cells into Th2 cells. Th2 cells continue to induce the immunoglobulin class switch from IgM to IgE. IgE antibodies bind to mast cells, inducing the release of lipid mediators, chemical and protein mediators, and pro-inflammatory cytokines (such as TNF-α, TGF-β, IL-4, and IL-13), leading to eczematous skin lesions (13).

Currently, several JAK inhibitors have been proven effective in treating AA, including tofacitinib, baricitinib, abrocitinib, and upadacitinib (14–17). The discovery and clinical application of these drugs provide new treatment options for adolescent patients with comorbid AA and AD. Existing studies show that the success rate of systemic JAK inhibitors in children is comparable to that in adults, with many patients showing significant responses; furthermore, only few non-responders and minimal side effects have been reported, including mild infections, diarrhea, and transient laboratory abnormalities. Broader research in fields such as rheumatology and oncology has been conducted on JAK inhibitors in pediatric patients, and phase 1 studies of JAK inhibitors for the treatment of pediatric malignant tumors and inflammatory diseases support the potential safety of these drugs and provide dosage guidance (18). However, for a better understanding of their therapeutic efficacy in skin diseases, more data from randomized controlled trials remain necessary.

Baricitinib has been approved in several countries for the treatment of pediatric atopic dermatitis and adult alopecia areata. In phase III clinical trials of baricitinib for adult severe alopecia areata, it was observed that some patients with extensive hair loss had a delayed response, and some patients with a history of more than 4 years did not respond to a treatment dose of 2 mg daily (19, 20). The dose needed to be increased to 4 mg daily to observe clinical effects. In the clinical study, there were no cases of venous thromboembolic events, arterial thrombotic events, major adverse cardiovascular events, gastrointestinal perforations, malignant tumors, tuberculosis, or confirmed opportunistic infections. Growth assessments indicated that the patient's growth rate was consistent with their baseline height, weight, or body mass index (BMI) percentiles. A similar situation was observed in this pediatric case. In our current clinical use of baricitinib and other JAK inhibitors, and even corticosteroids, in adult patients, some have experienced hair loss again after discontinuing the medication. This means that current drugs cannot completely control the follicular inflammation in such patients, and further basic research is needed to substantiate this.

## Data Availability

The original contributions presented in the study are included in the article/Supplementary Material, further inquiries can be directed to the corresponding authors.
