# Supplementary material for: Case Report: Baricitinib improved alopecia areata in a pediatric patient with atopic dermatitis
Source: Front Pediatr. 2025 Jan 10;12:1497285. doi: 10.3389/fped.2024.1497285 (PMC11760602; doi:10.3389/fped.2024.1497285)
Supplement: Supplementary file 1 [file Datasheet1.pdf]

Charlesworth
Author Services

EDITORIAL CERTIFICATE

This document certifies that the manuscript below was edited for correct English language usage, grammar, punctuation and spelling by qualified native English speaking editors at Charlesworth Author Services.

Paper Title:

Baricitinib improved alopecia areata in a pediatric patient with atopic dermatitis: A case report

Author:

Sihan Wang

Date certificate issued:

October 25, 2024

cwauthors.com
