# Supplementary figures and images for: Case Report: Baricitinib improved alopecia areata in a pediatric patient with atopic dermatitis
Source: Front Pediatr. 2025 Jan 10;12:1497285. doi: 10.3389/fped.2024.1497285 (PMC11760602; doi:10.3389/fped.2024.1497285)

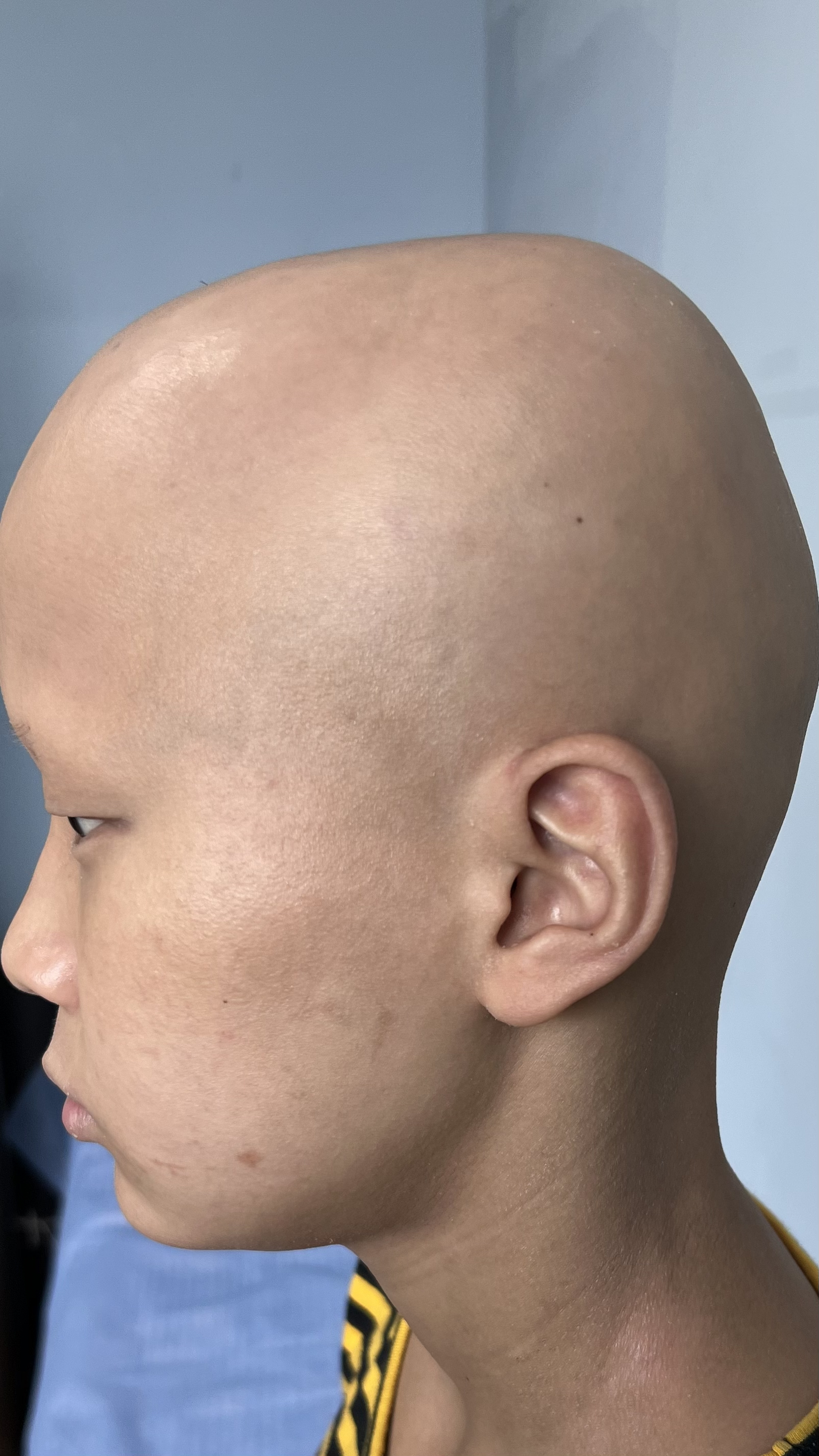

Supplement: Supplementary file 3 [file Image1.jpeg]

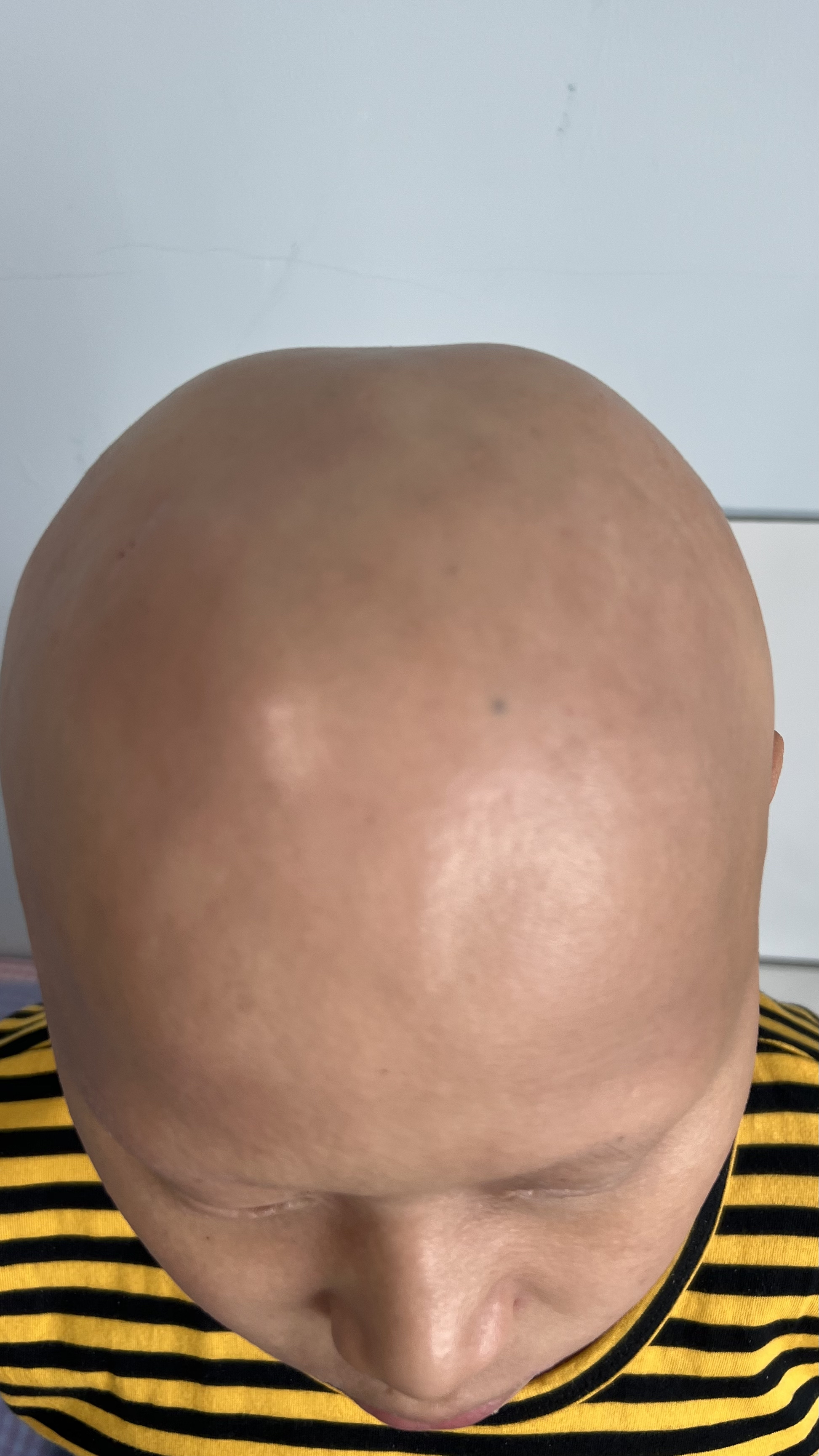

Supplement: Supplementary file 4 [file Image2.jpeg]

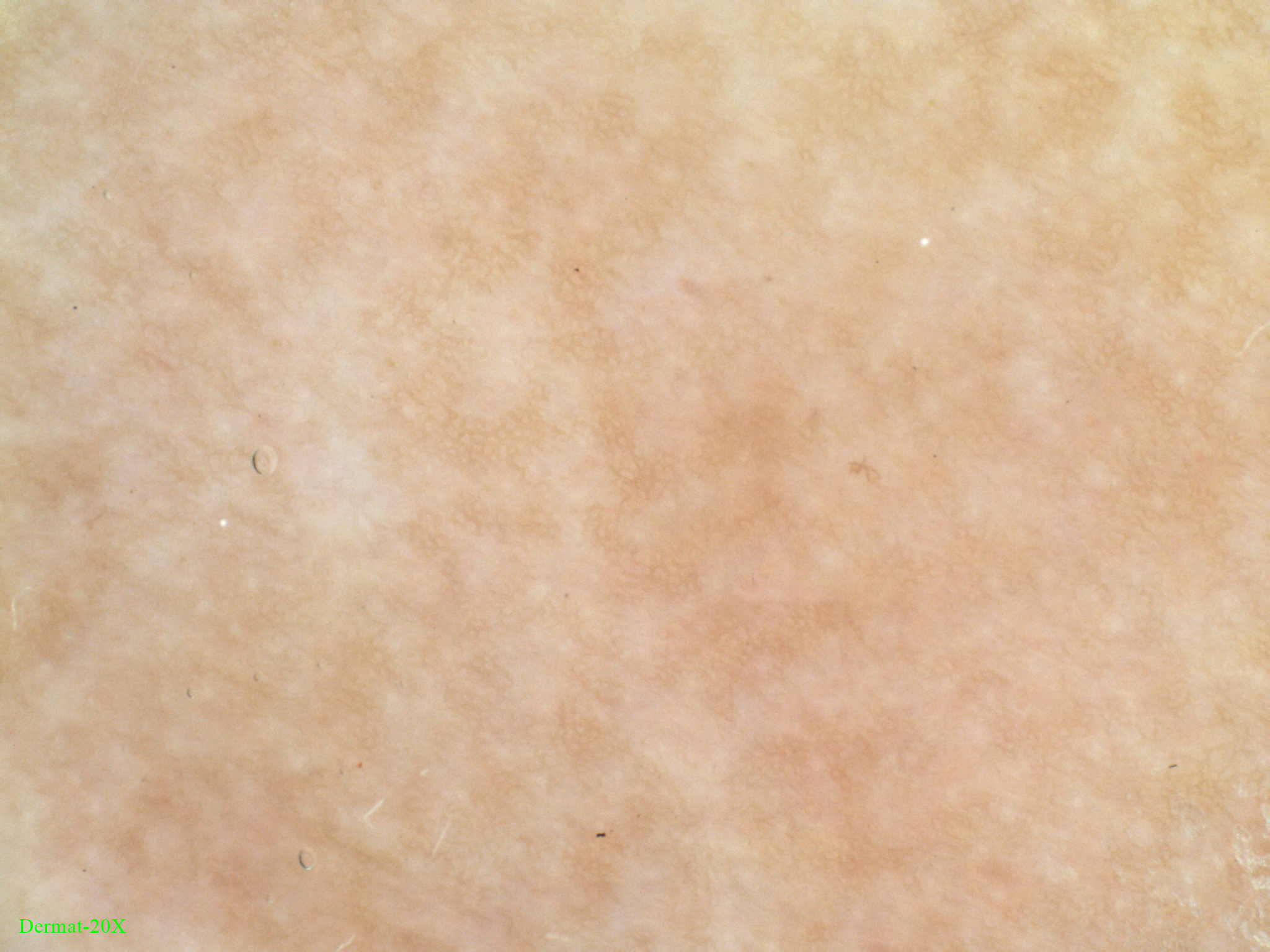

Supplement: Supplementary file 5 [file Image3.jpeg]

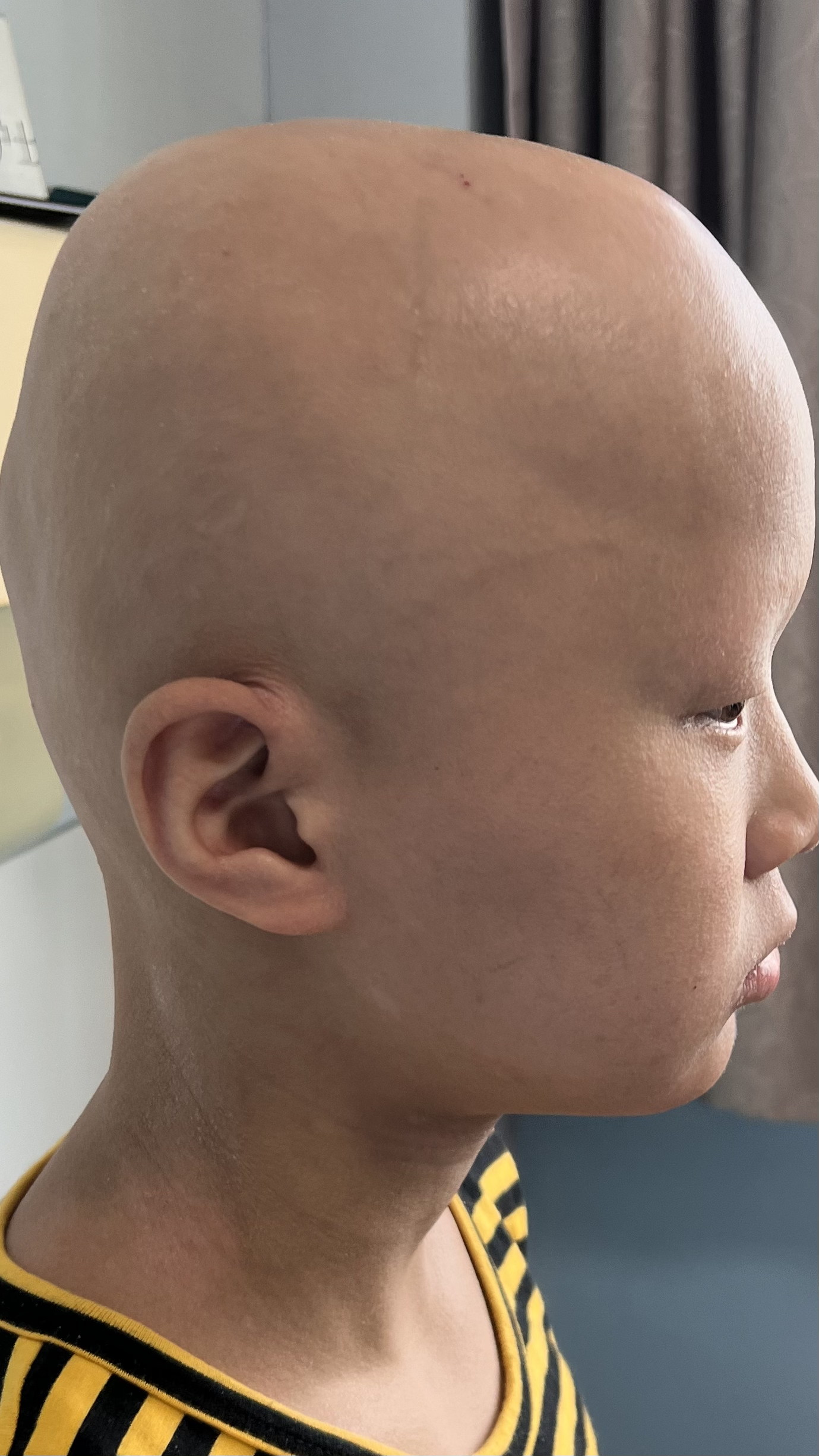

Supplement: Supplementary file 6 [file Image4.jpeg]

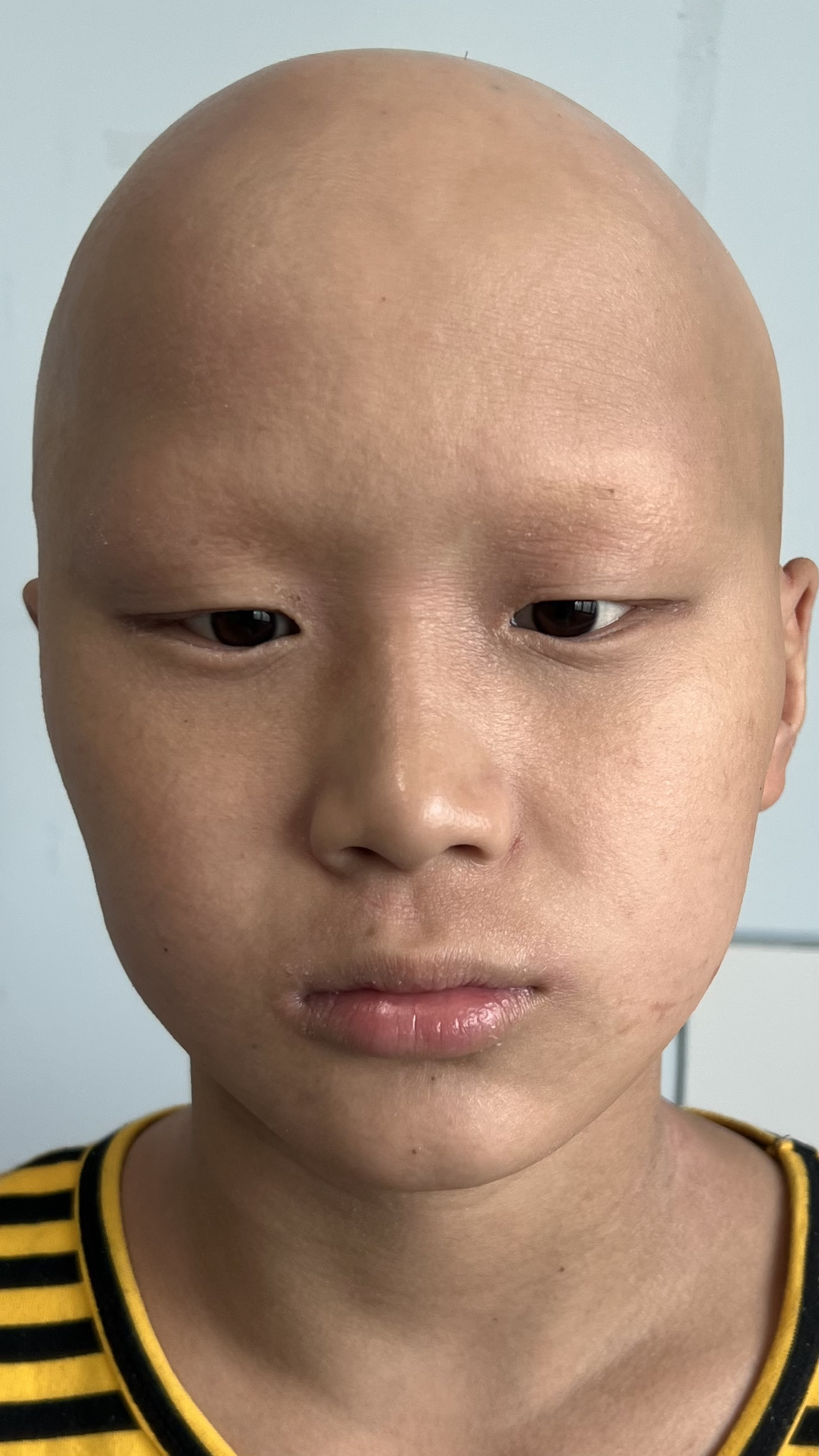

Supplement: Supplementary file 8 [file Image6.jpeg]

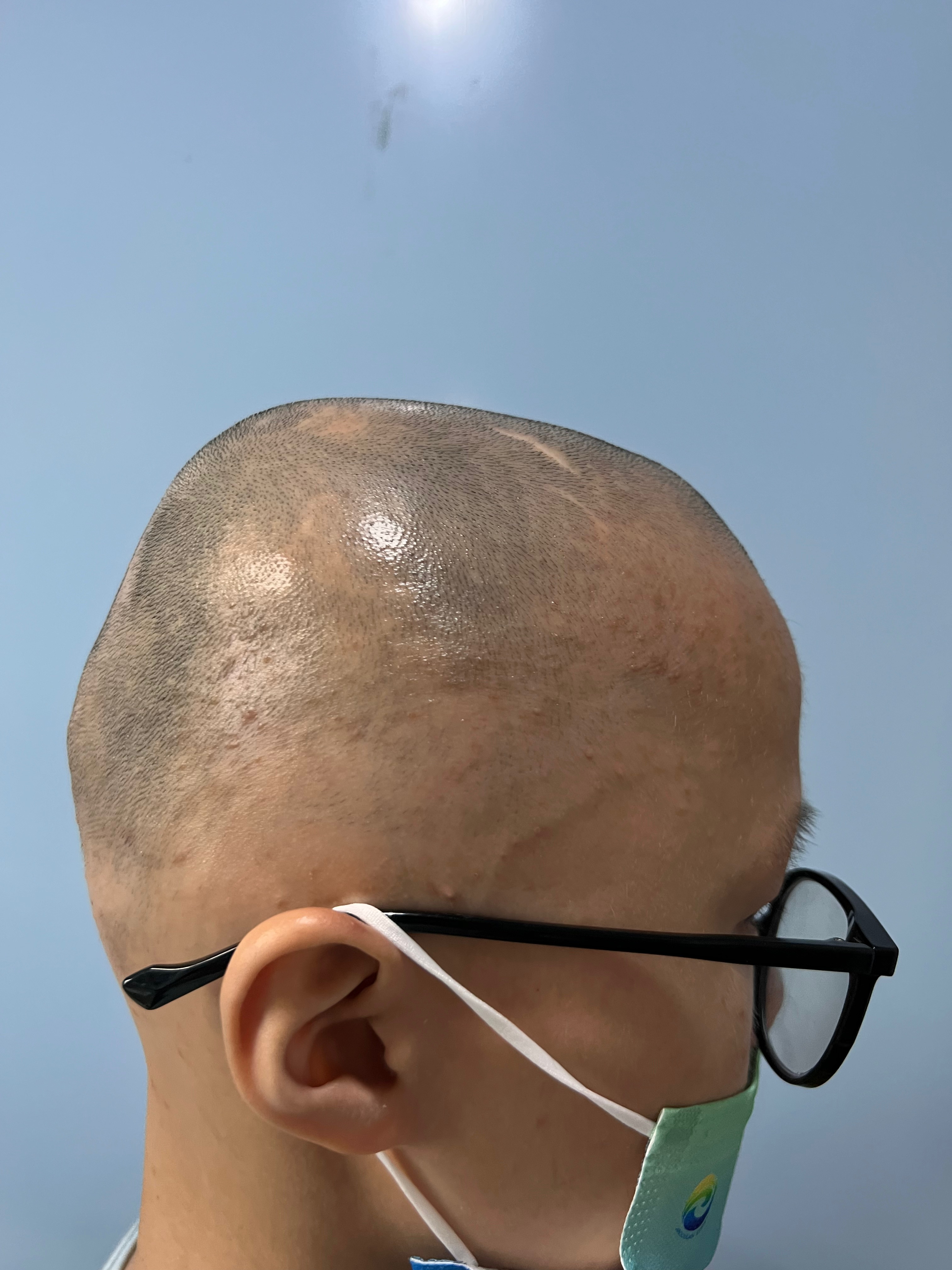

Supplement: Supplementary file 9 [file Image7.jpeg]

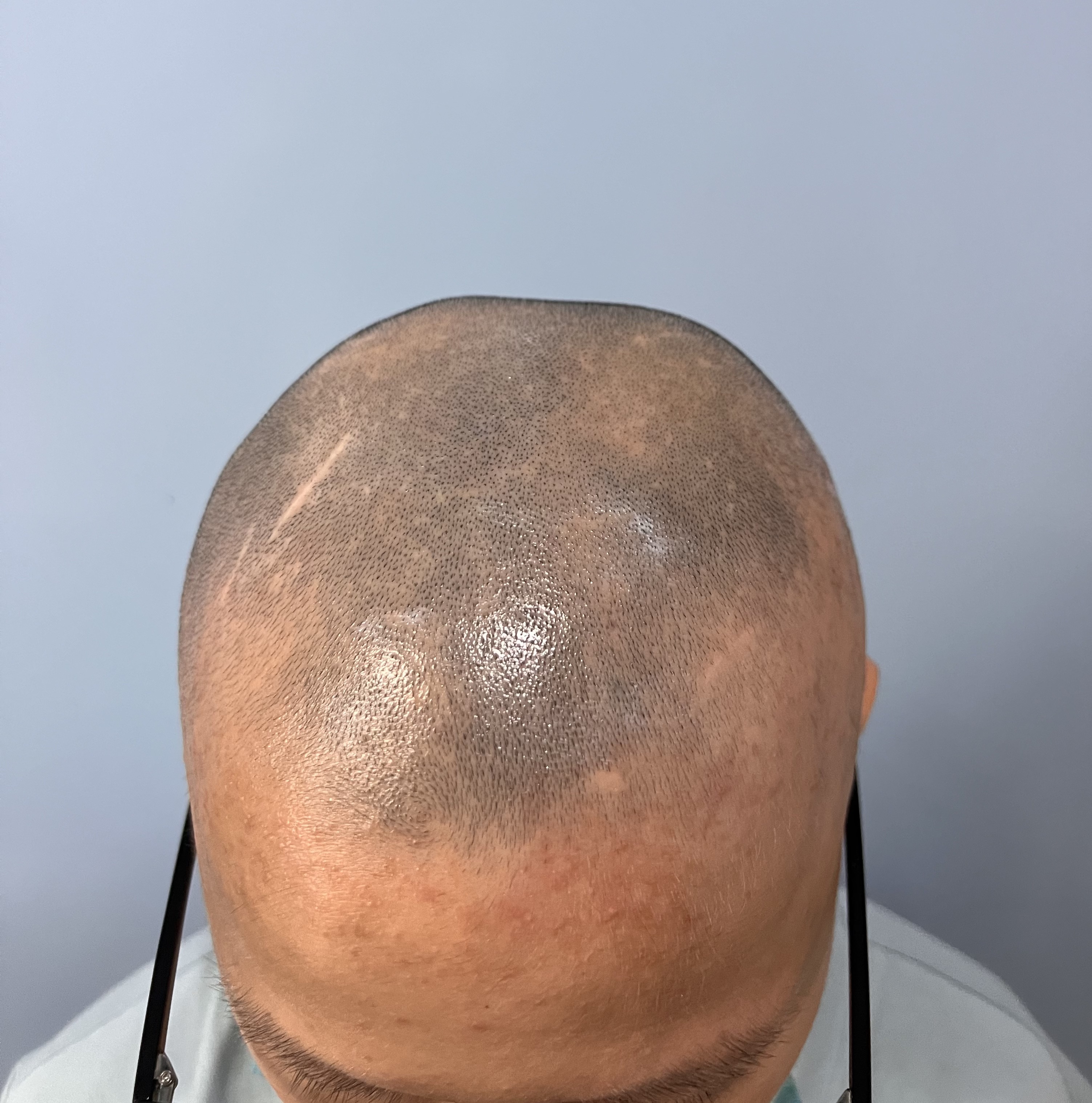

Supplement: Supplementary file 10 [file Image8.jpeg]

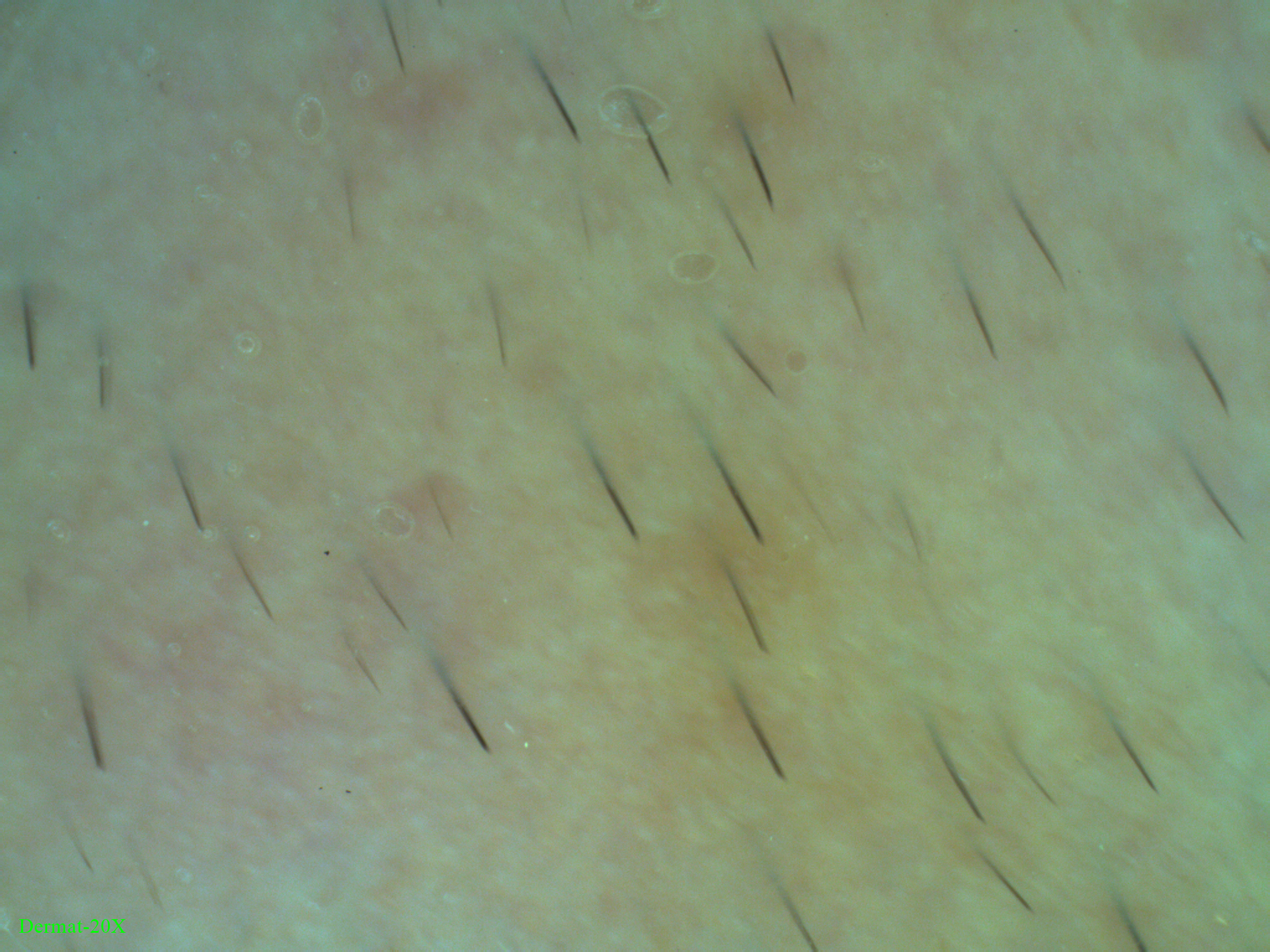

Supplement: Supplementary file 11 [file Image9.jpeg]

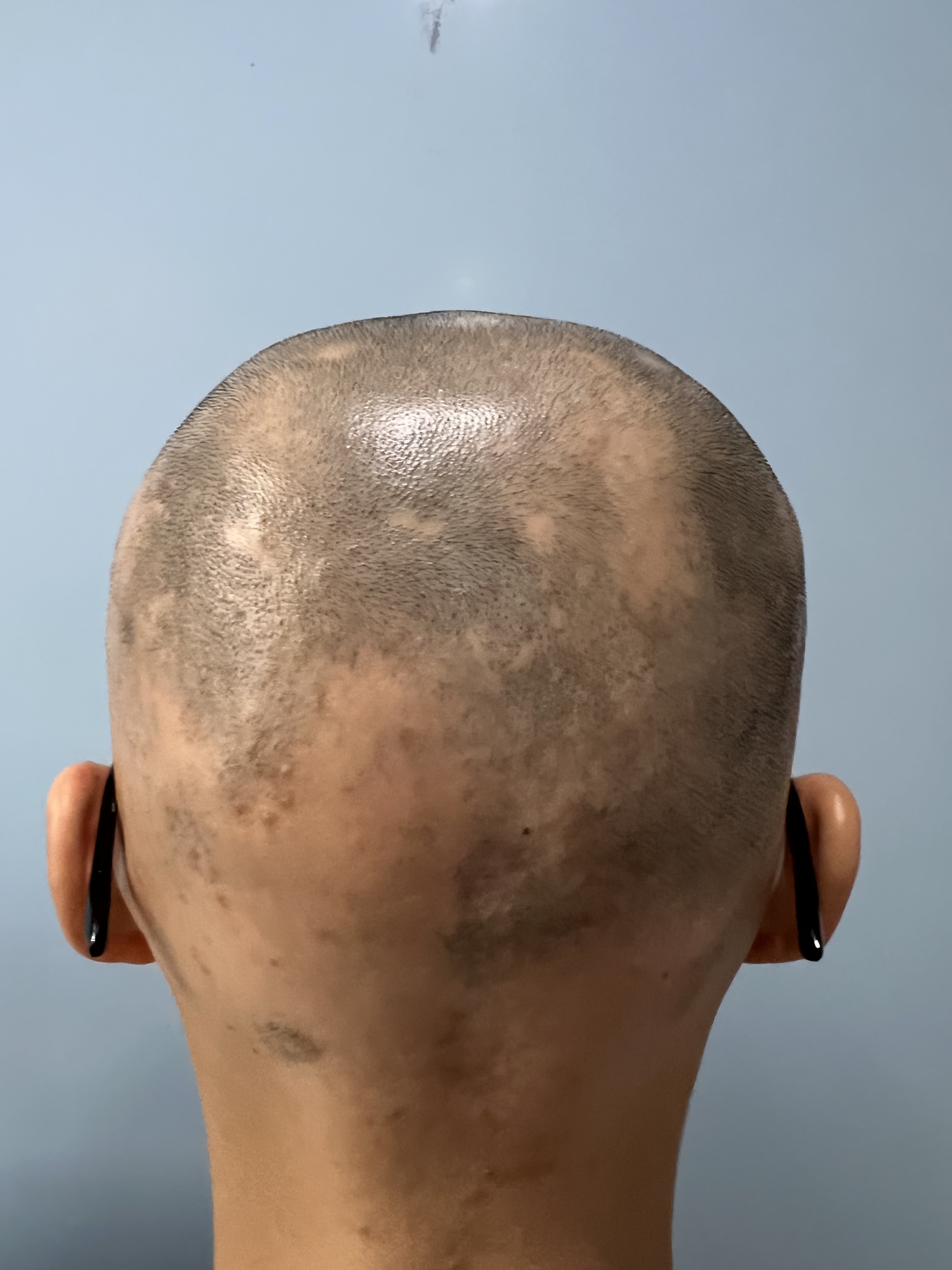

Supplement: Supplementary file 12 [file Image10.jpeg]

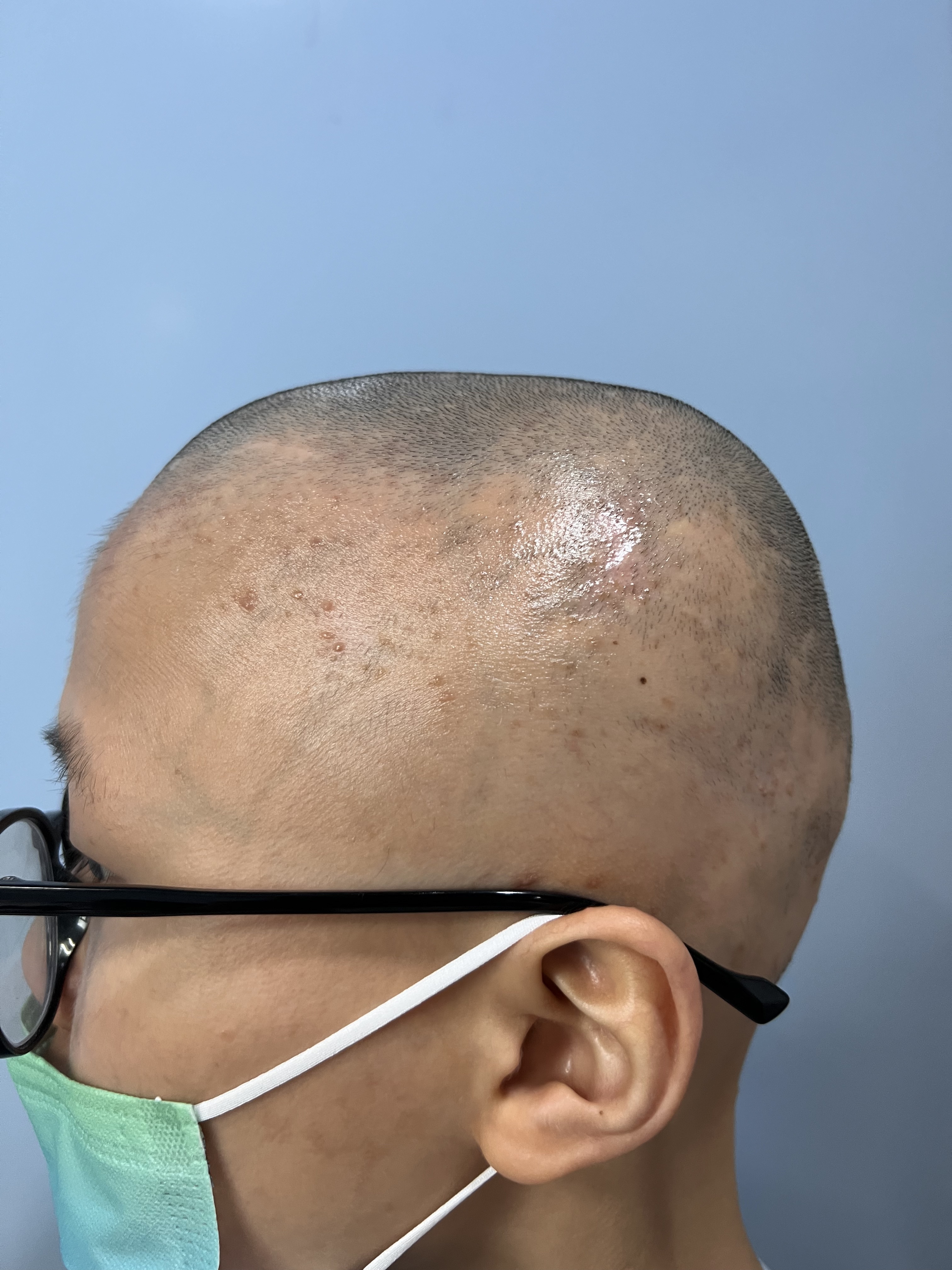

Supplement: Supplementary file 13 [file Image11.jpeg]

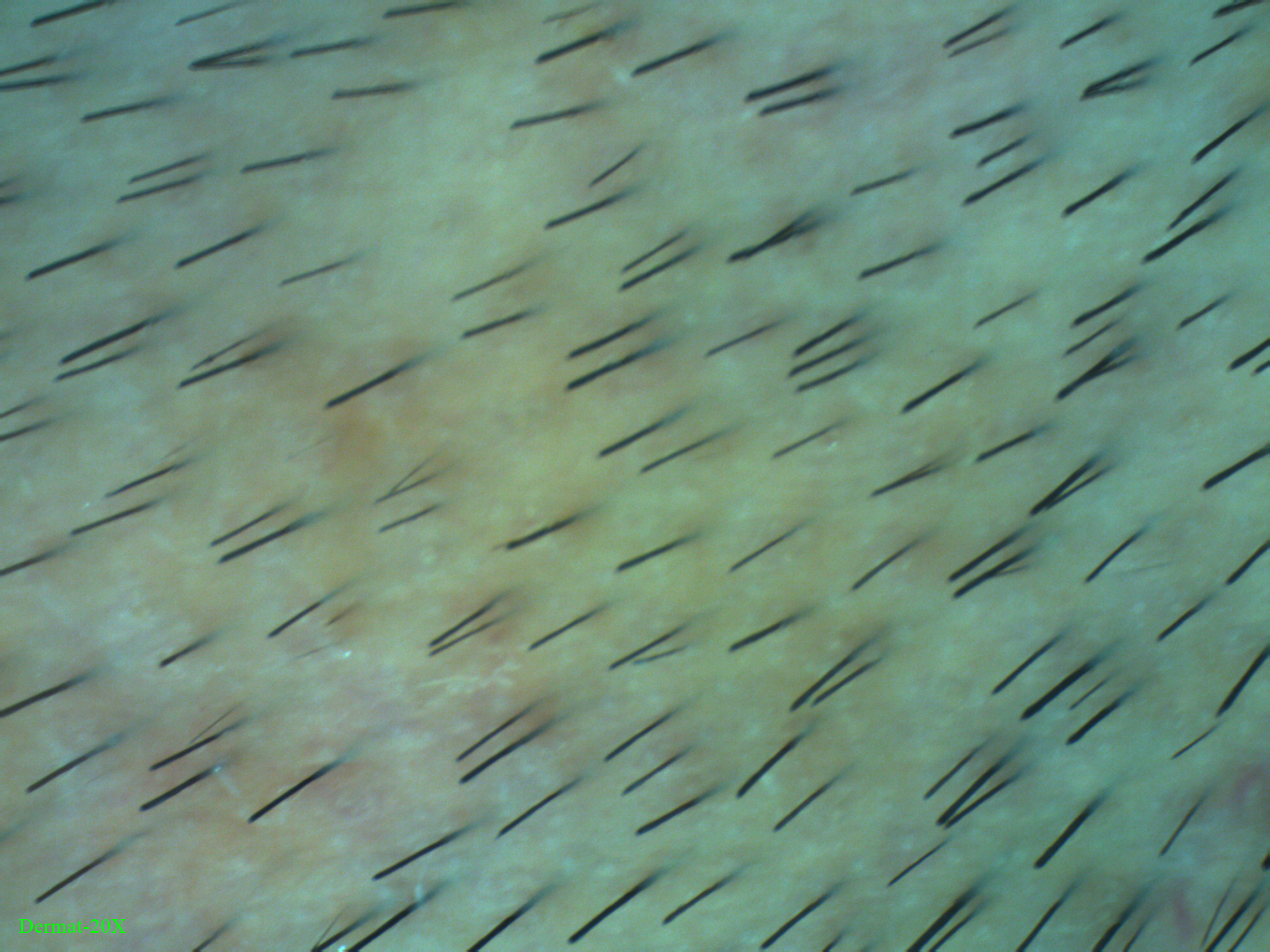

Supplement: Supplementary file 14 [file Image12.jpeg]

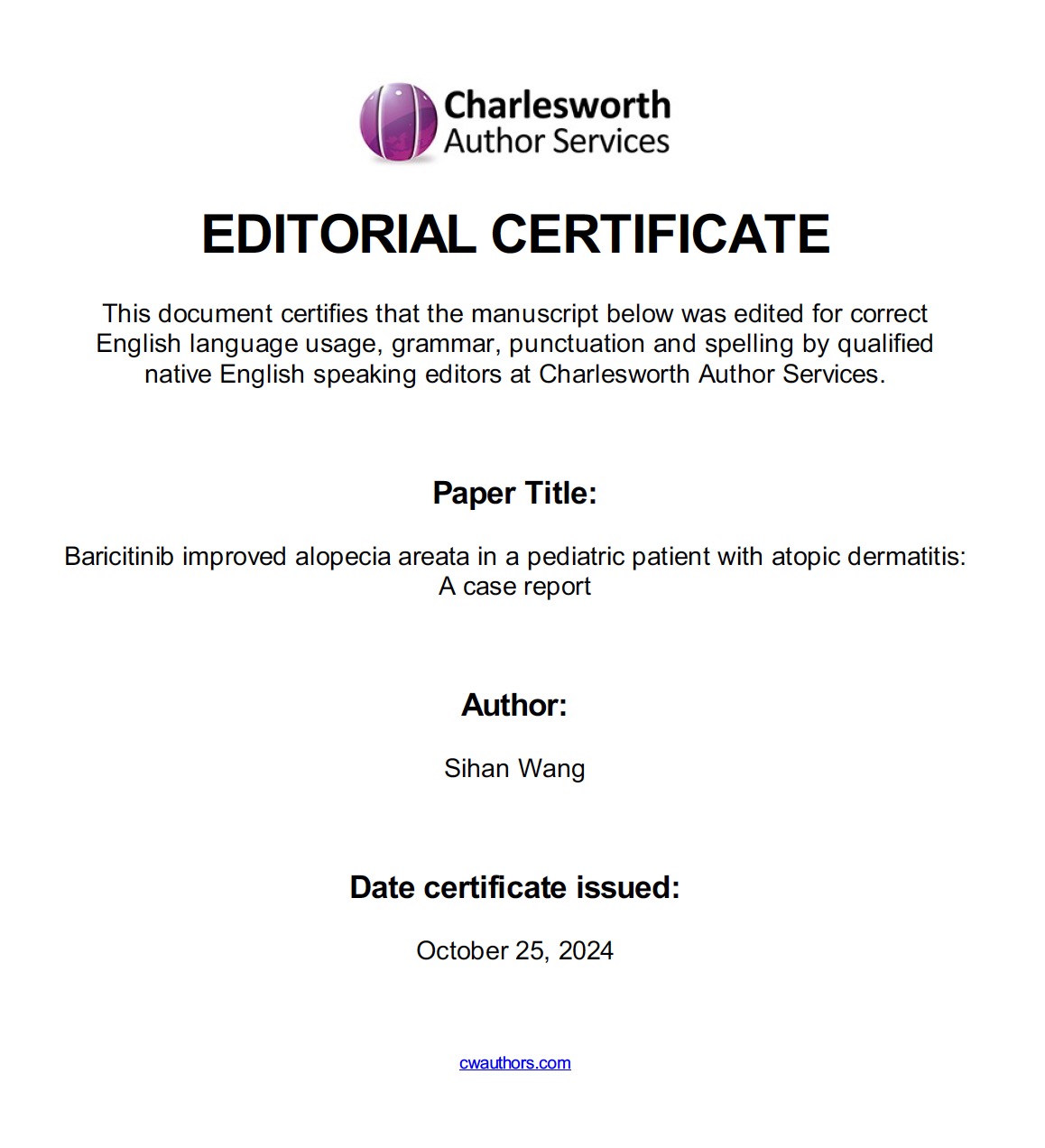

Supplement: Supplementary file 15 [file Image13.jpeg]

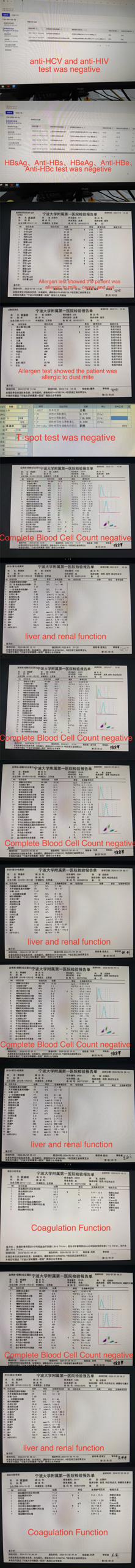

Supplement: Supplementary file 16 [file Image14.jpeg]
